# CRISPR/Cas9-Mediated *TARDBP* Knockout Reduces Triacylglycerol Content and Key Milk Fat Metabolism Gene Expression in MAC-T Cells

**DOI:** 10.3390/ani15172607

**Published:** 2025-09-05

**Authors:** Yaran Zhang, Qinglan Zhang, Yaping Gao, Yao Xiao, Jinpeng Wang, Chunhong Yang, Zhihua Ju, Xiaochao Wei, Xiuge Wang, Qiang Jiang, Jinming Huang

**Affiliations:** 1 Key Laboratory of Livestock and Poultry Multi-omics of Ministry of Agriculture and Rural Affairs, Institute of Animal Science and Veterinary Medicine, Shandong Academy of Agricultural Sciences, Jinan 250100, China; zhang_ya_ran@126.com (Y.Z.);; 2 Shandong Technology Innovation Center of Dairy Cattle Breeding Industry, Jinan 250100, China

**Keywords:** bovine milk fat, *TARDBP*, CRISPR/Cas9, transcriptome sequencing

## Abstract

TARDBP mediates milk fat secretion in mice by binding to UG-rich sequences in the 3′ untranslated region (UTR) of *BTN1A1* and *XDH* mRNA and enhancing their mRNA stability. However, its role in bovine milk lipid metabolism remains unclear. To investigate this, we generated *TARDBP* knockout (KO) MAC-T cells using CRISPR/Cas9 technology, quantified triacylglycerol (TAG) levels in both cells and culture supernatant, and examined the impact of *TARDBP* on mRNA levels in MAC-T cells through transcriptome sequencing. We found that deletion of *TARDBP* reduced TAG content in both MAC-T cells and the supernatant, as well as decreased mRNA levels of *CD36*, *FABP4*, *DGAT1*, *PPARG*, and *PPARGC1A*. However, the expression of *BTN1A1* and *XDH* was unaffected in bovine MAC-T cells. Sequence analysis further revealed TG-rich sequences within bovine *PPARG* and *PPARGC1A* but not in *FABP4*, *DGAT1*, *CD36*, or *BTN1A1* and *XDH*. These findings suggest that *TARDBP* may regulate bovine lipid metabolism through a mechanism distinct from that described in mice. This study provides new insights into the molecular role of *TARDBP* in bovine milk fat metabolism and establishes a foundation for understanding its contribution to dairy cattle breeding and milk quality improvement.

## Simple Summary

Milk fat content in cows is important for dairy quality, but how it is regulated is not fully understood. In mice, a protein called TARDBP (trans-activating response region DNA-binding protein) controls milk fat by stabilizing certain genes, but its role in cows was unknown. To investigate this, we used gene-editing technology to remove TARDBP from cow mammary cells and studied its effects. We found that cells without TARDBP produced less fat both inside the cells and in their secretions. They also showed reduced mRNA levels of several key fat-related genes, including *CD36*, *FABP4*, *DGAT1*, *PPARG*, and *PPARGC1A*. Unlike in mice, TARDBP did not affect the same genes (*BTN1A1* and *XDH*) in cows. Instead, our results suggest that TARDBP likely binds to specific regions of the *PPARG* and *PPARGC1A* genes, influencing their stability and, in turn, regulate other fat-related genes. These findings reveal a new way that TARDBP regulates milk fat in cows, differing from its role in mice. This research helps us better understand how milk fat production is controlled in dairy cows, which could lead to improved breeding strategies for higher-quality milk. Further studies are needed to uncover the exact mechanisms involved.

## 1. Introduction

Milk fat content is an economically important trait in the dairy industry, as it influences the flavor, texture and chemical–physical characteristics of milk and dairy products [1]. In general, higher milk fat content is indicative of superior raw milk quality [2]. In many countries, milk fat content serves as a key determinant of raw milk pricing [2]. Consequently, improving milk fat content is a major goal in dairy breeding programs, particularly in Holstein cows.

Milk fat content is a complex quantitative trait influenced by both genetic and environmental factors [3,4]. In bovine mammary epithelial cells (MECs), the synthesis and secretion of TAG are crucial physiological processes that determine milk fat content. At the molecular level, these processes require the coordinated action of numerous enzymes and proteins. For instance, acyl-CoA synthetase short-chain family members (ACSSs), fatty acid synthase (FASN), and acetyl-CoA carboxylase alpha (ACACA) catalyze de novo synthesis of fatty acids (FAs); CD36 molecule (CD36), lipoprotein lipase (LPL), fatty acid transport proteins (FATPs), fatty acid binding proteins (FABPs), and acyl-CoA synthetase long-chain family members (ACSLs) mediate FA uptake, activation, and transport; elongation of very long chain fatty acid proteins (ELOVLs), stearoyl-CoA desaturases (SCDs), and fatty acid desaturases (FADSs) regulate FA elongation and desaturation; meanwhile, glycerol-3-phosphate acyltransferase, mitochondrial (GPAM), 1-acylglycerol-3-phosphate O-acyltransferases (AGPATs), diacylglycerol O-acyltransferases (DGATs), and phosphatidate phosphatase (LPIN) are responsible for catalyzing triacylglycerol (TAG) synthesis; proteins such as butyrophilin, subfamliy 1, member A1(BTN1A1), xanthine dehydrogenase (XDH) and perilipins (PLINs) are further involved in milk lipid droplet formation and secretion [5,6,7]. The expression of these enzymes and proteins is controlled by complex gene networks. Transcriptional regulators including sterol regulatory element-binding protein 1 (SREBP1), peroxisome proliferator-activated receptor gamma (PPARG), PPARG coactivator 1 alpha (PPARGC1A), and insulin induced gene 1 (INSIG1) play pivotal roles in milk fat metabolism [5,6,7]. In addition, other genes such as eukaryotic translation elongation factor 1 delata gene (*EEF1D*) [8] and collagen type VI alpha 1 chain gene (*COL6A1*) [9], as well as non-coding RNAs including Lnc-TRTMFS (transcripts related to milk fat synthesis) [10], miR-200a [11] and miR-224 [12] have been successively identified as regulators of milk fat metabolism. Despite these advances, the gene networks governing bovine milk fat metabolism remain incompletely understood.

TARDBP (trans-activating response region DNA-binding protein), also known as TDP-43 and encoded by the *TARDBP* gene, is a multifunctional DNA/RNA-binding protein [13]. By binding to long TG/UG-rich sequences in target genes, it regulates gene expression in various ways, including modulating RNA processing, stability, and translation, as well as DNA transcription [13,14,15,16,17,18]. *TARDBP* is broadly expressed in human and rodent tissues [19] and participates in diverse biological processes, such as embryonic development [20], mammary gland development [21], fat metabolism [22,23,24], viral infection [25,26], and neurological disorders [27,28,29]. In fat metabolism, conditional *TARDBP* KO mice induced with tamoxifen die rapidly, displaying dramatic fat loss, increased fatty acid consumption, and downregulation of the obesity-associated gene *TBC1D1*, resulting in severe disruption of the body’s fat metabolism [22]. Conversely, transgenic mice overexpressing *TARDBP* develop marked obesity characterized by excessive fat deposition and adipocyte hypertrophy compared with non-transgenic littermates [23]. *TARDBP* has also been implicated in the obesity pathogenesis [30]. Additionally, in the mammary gland, conditional *TARDBP* KO in mice causes failure of milk lipid secretion by affecting the mRNA stability of *BTN1A1* and *XDH* [24]. Despite these findings in rodents, the role of *TARDBP* in bovines has not yet been investigated.

The objective of this study was to investigate the role of bovine *TARDBP* in milk fat metabolism. To this end, a *TARDBP* KO bovine mammary epithelium cell line (MAC-T) was generated using CRISPR/Cas9 technology. Loss of *TARDBP* significantly reduced TAG content in both MAC-T cells and the culture supernatant. Transcriptome sequencing further revealed that *TARDBP* deficiency led to downregulation of key milk fat metabolism genes, including *CD36*, *FABP4*, *DGAT1*, *PPARG*, and *PPARGC1A*. Notably, TG-rich sequences were identified in both *PPARG* and *PPARGC1A* genes, suggesting a direct regulatory role of TARDBP on these genes. However, diverging from results observed in mice, no significant changes were observed in the expression of bovine *BTN1A1* and *XDH*, and no TG-rich sequences were detected within these two genes, indicating the existence of certain species divergence in the molecular regulation of milk fat metabolism. Collectively, this study provides the first evidence of *TARDBP*’s role in bovine milk fat metabolism and uncovers its functional divergence between cattle and mice. These findings provide a foundation for further investigations into the molecular regulatory mechanisms of milk fat metabolism and may inform genetic breeding strategies for improved dairy fat traits.

## 2. Materials and Methods

### 2.1. Guide RNA (gRNA) Design and CRISPR/Cas9 Vector Construction

A gRNA sequence (5′-GAGATACCATCGGAAGACGA-3′) targeting exon 2 of the bovine *TARDBP* gene (GenBank accession: NC_037343) was designed using the online tool E-Crisp (www.e-crisp.org/E-CRISP/designcrispr.html, accessed on 19 September 2022), and potential off-target sites were assessed via BLAST/BLAT in Ensembl (http://asia.ensembl.org/Bos_taurus/Tools/Blast, accessed on 1 September 2025). A *Bbs*I restriction site was added to the 5′ end of the gRNA sequence, and complementary forward and reverse oligos were synthesized. Following annealing, the gRNA oligos were cloned into the Genloci pGK1.1(Puro^r^) vector (Genloci Biotech, Nanjing, China), which contains both a Cas9 expression cassette and a gRNA cloning cassette, hereafter referred to as pGK1.1. The resulting recombinant vector was designated pGK1.1-gRNA.

### 2.2. Cell Culture, Transfection and Screening for TARDBP KO Cells

MAC-T, an immortalized bovine mammary epithelial cell line, was a generous gift from Professor Kerong Shi at Shandong Agricultural University. The cells were cultured in basal medium (DMEM/F12 (C11330500BT, Gibco, Grand Islang, NY, USA), supplemented with 10% FBS (A5669701, Gibco) and 100 U/mL of penicillin/streptomycin (15140122, Gibco) at 37 °C under 5% CO_2_. The pGK1.1-gRNA vectors were transfected into MAC-T cells using Lipofectamine 3000 (L3000008, Invitrogen, Grand Island, NY, USA) according to the manufacturer’s instructions. After transfection for 48 h, cells were cultured in fresh basal medium for an additional 48 h. To select for successfully transfected cells, cultures were maintained in drug selection medium, the puromycin-containing medium (DMEM/F12, 10% FBS, 100 U/mL of penicillin/streptomycin, 3 μg/mL of puromycin), for 5 days, with medium replacement every 2 days. Cells were then returned to basal medium and cultured for 24 h prior to downstream assays.

Before monoclonal *TARDBP* knockout (KO) cell isolation, the editing activity of the pGK1.1-gRNA vector was assessed by PCR amplification and Sanger sequencing of the target region using genomic DNA extracted from the transfected cell pool post-drug selection. Wild-type (WT) MAC-T cells served as controls. Briefly, trypsinized MAC-T cells were collected (~25% of the population), and genomic DNA was extracted using a commercial kit (DP304-03, TIANGEN, Beijing, China) according to the manufacturer’s instructions. Using the extracted DNA as template, a 651 bp DNA region spanning the target site was amplified using the primers: Forward: 5′-GCCTTTACTTTCTCGTTT-3′, Reverse: 5′-CTTGGCATTAGTTCAGCA-3′. The PCR protocol involved an initial denaturation at 95 °C for 5 min, followed by 35 cycles of 94 °C for 30 s, 50.4 °C for 30 s, 72 °C for 50 s, with a final extension at 72 °C for 10 min. PCR products were subjected to Sanger sequencing. The sequencing chromatogram of PCR products from the puromycin-selected, pGK1.1-gRNA-transfected cell pool showed overlapping peaks at the target site within the bovine *TARDBP* gene (Figure S1), whereas no such overlapping peaks were observed in WT controls, confirming successful CRISPR/Cas9-mediated gene editing via the pGK1.1-gRNA vector. Based on this qualitative confirmation, we proceeded to screen for *TARDBP* KO cells.

To isolate *TARDBP* KO cells, the drug-selected mixed MAC-T cells were digested with trypsin, resuspended at a density of 2–5 cells/mL, and seeded into 96-well plates. After sufficient proliferation, cells were transferred to 48-well plates and cultured to ~90% confluence. Subsequently, genomic DNA was extracted from ~40–50% of the cells in each well, according to methods mentioned above. And using the extracted DNA as templates, *TARDBP* KO cells were identified by PCR amplification and sequencing of the same 651 bp DNA target region, using the primers and PCR conditions described above.

### 2.3. Western Blot

Total protein was extracted from MAC-T cells as follows. Briefly, after trypsinization, cells were collected by centrifugation and washed twice with Dulbecco’s phosphate-buffered saline (DPBS). Cells were then lysed in pre-chilled RIPA buffer (R0010, Solarbio, Beijing, China) containing protease inhibitor cocktail (S8820-2TAB, Roche Diagnostics, Basel, Switzerland), and sonicated on ice three times for 4 s each. Lysates were incubated on ice for 30 min, followed by centrifugation at 12,000 rpm for 15 min at 4 °C. The supernatants were mixed with protein loading buffer and denatured at 95 °C for 10 min.

Denatured protein lysates were separated by SDS-PAGE and transferred onto a nitrocellulose membrane via electroblotting. The membrane was then blocked with 5% (w/v) skim milk for 1 h at room temperature. After being washed three times with PBS for 5 min, according to the molecular weight of protein bands, the membrane was cut in half and respectively incubated overnight at 4 °C with primary antibodies: mouse anti-TARDBP (1:5000; NBP1-92695SS, Novus Biologicals, Centennial, CO, USA) and mouse HRP-conjugated anti-GAPDH (1:10,000; HRP-60004, proteintech, Wuhan, China). After three washes with PBS for 5 min, the membrane incubated with TARDBP antibody was further incubated with HRP-conjugated goat anti-mouse IgG (1:10,000; AS003, ABclonal, Wuhan, China) for 1 h at room temperature. Meanwhile, the GAPDH-bound membrane was soaked in PBS and waited for development. And, then, the membrane bound with TARDBP antibody and HRP-labeled secondary antibodies was washed three times with PBS for 5 min. Finally, protein bands were visualized using enhanced chemiluminescence reagent (1810202, CLINX, Shanghai, China) and imaged with a ChemiScope 6200 system (CLINX, Shanghai, China). Western blot analysis was performed independently three times with consistent results.

### 2.4. cDNA Library Construction and Transcriptome Sequencing

WT and *TARDBP* KO MAC-T cells were seeded into 6-well plates, with five replicate wells per genotype (*n* = 5). The cells were cultured in lactogenesis induction medium supplemented with 5 μg/mL of bovine insulin (I5500, Sigma, St. Louis, MO, USA), 1 μg/mL of hydrocortisone (S31360-5 g, Yuanye, Shanghai, China), and 2.5 μg/mL of prolactin (PRS-CYT-240-20 μg, ProSpec, Ness Ziona, Israel) in the basal medium for 48 h prior to subsequent experiments.

Total RNA was isolated from both WT and *TARDBP* KO cells (*n* = 5) using TRNzol Universal Reagent (DP424, TIANGEN). All procedures were performed on ice using RNase-free consumables. Briefly, cells in 6-well plates were washed twice with PBS, lysed with 1 mL of TRNzol Universal Reagent per well at room temperature for 5 min with gentle pipetting, and the lysates were then transferred to centrifuge tubes. Subsequently, 200 μL of chloroform was added to each tube, and tubes were shaken vigorously for 15 s, incubated at room temperature for 3 min, and centrifuged at 12,000 rpm for 15 min at 4 °C. The colorless upper aqueous phase containing RNA was carefully transferred to a new RNase-free tube without disturbing the interphase. RNA was precipitated by adding an equal volume of isopropanol to the supernatant, mixed gently, incubated for 10 min at room temperature, and centrifuged at 12,000 rpm for 10 min at 4 °C. After discarding the supernatant, the RNA pellet was washed with 1 mL 75% ethanol by gentle inversion, centrifuged at 10,000 rpm for 5 min at 4 °C, and the ethanol supernatant was carefully discarded. Finally, the RNA pellet was air-dried for 3 min at room temperature and dissolved in 35 μL of RNase-Free ddH_2_O. RNA integrity was assessed using the RNA Nano 6000 Assay Kit on a Bioanalyzer 2100 system (Agilent Technologies, Santa Clara, CA, USA).

mRNA was purified from total RNA using poly-T oligo-attached magnetic beads, followed by fragmentation with divalent cations at elevated temperature in the First Strand Synthesis Reaction Buffer. First-strand cDNA was then synthesized using random hexamer primers and M-MuLV reverse transcriptase. Subsequently, the mRNA template was degraded with RNase H, and second-strand cDNA was synthesized using DNA polymerase I. After terminal repair, 3′ ends adenylation, and ligation of adaptors for sequencing, cDNA fragments of 370–420 bp were selected using AMPure XP beads (A63880, Beckman Coulter, IN, USA). PCR amplification was performed, and the cDNA library was obtained after purifying the PCR products using AMPure XP beads.

After the library passed inspection using the Bioanalyzer 2100 system, the cDNA libraries were sequenced on an Illumina NovaSeq X plus platform, generating 150 bp paired-end raw reads (raw data). Raw reads in fastq format were filtered to remove adapter-containing reads, reads with poly-N (undetermined base information), and low-quality reads. The sequencing error rates (Q20 and Q30) and GC content were then assessed to evaluate sequencing quality. Finally, high-quality clean data were obtained for subsequent analyses.

### 2.5. Reads Mapping to the Reference Genome and Differential Expression Analysis

The index of the *Bos taurus* reference genome ARS-UCD1.3 (GenBank assembly accession: GCF_002263795.3; https://www.ncbi.nlm.nih.gov/datasets/genome/GCF_002263795.3/, accessed on 1 September 2025) was built, and clean reads were aligned to the reference genome using HISAT2 (v2.0.5) software [31]. Mapped reads from each sample were assembled using StringTie software (v1.3.3b) [32], and the number of reads mapped to each gene was quantified using featureCounts software (v1.5.0-p3) [33]. Then, gene expression levels were calculated as FPKM (expected number of Fragments Per Kilobase of transcript sequence per Millions base pairs sequenced), considering both gene length and the number of reads mapped to each gene. Differential expression analysis between WT and *TARDBP* KO cells was performed using DESeq2 software (v1.20.0) [34]. To control the false discovery rate, *p*-values were adjusted using the Benjamini and Hochberg method. Genes with an adjusted *p*-value < 0.05 and |log2FoldChange| > 1 were considered differentially expressed.

### 2.6. Gene Ontology (GO) and Kyoto Encyclopedia of Genes and Genomes (KEGG) Enrichment Analysis

To investigate the potential roles of differentially expressed genes (DEGs) between WT and *TARDBP* KO cells in cellular functions and biological processes, GO and KEGG enrichment analyses of the DEGs were performed using clusterProfiler (v 3.8.1) package in R, which corrected for gene length bias. GO terms and KEGG pathways with an adjusted *p*-value < 0.05 were considered statistically significant. The data were visualized using ggplot2 package in R.

### 2.7. Quantitative Real-Time PCR (qRT-PCR) Validation

To validate the transcriptome sequencing results, four DEGs were randomly se-lected for qRT-PCR analysis, along with three downregulated genes associated with milk fat metabolism. Total RNA was extracted from MAC-T cells using the TRIzol method as described above, and cDNA was reverse-transcribed with the PrimeScript^TM^ RT reagent Kit with gDNA eraser (RR047A, TaKaRa, Dalian, China) following the manufacturer’s instructions. qRT-PCR was performed on a LightCycler 480 system (Roche, Mannheim, Germany) using TB Green^®^ Premix Ex Taq™ II (RR820A, TaKaRa, Dalian, China) under a two-step PCR protocol following the manufacturer’s guidelines. Primers used for bovine genes are listed in Table 1. Relative mRNA expression levels were normalized to *β*-actin and quantified using the 2^−△△Ct^ method. All qRT-PCR assays included five biological replicates, each measured in triplicate technical replicates.

### 2.8. Analyses of TARDBP Binding Motif of Bovine Genes

Given that TARDBP regulates RNA metabolism by binding to long TG-rich DNA or UG-rich RNA sequences, the DNA sequences of downregulated genes involved in milk fat metabolism were analyzed to identify potential TARDBP binding motifs. Specifically, the target bovine gene sequences were downloaded from GenBank. We searched for TG-rich motifs, which comprise more than six consecutive TG repeats [13], using a custom Python (v 3.8.10) script based on regular expression matching. Both sense and antisense strands were scanned, and the analysis was applied to the 5′ UTR, 3′ UTR, exons, introns, and the promoter region (2000 bp upstream of the transcription start site) of each gene.

### 2.9. Detection of TAG Content

TAG content in both MAC-T cells and the culture supernatant of different genotypes was measured using commercial kits (E1003, E1013, Applygen Technologies Inc., Beijing, China) as previously described [35,36]. Briefly, WT and *TARDBP* KO cells were seeded in 6-well plates, with three replicate wells per genotype (*n* = 3) and cultured in lactogenesis induction medium for 48 h as mentioned above. The cell-free supernatant was collected, and cells in each well were harvested by trypsinization followed by centrifugation at 1000 rpm for 5 min. And then, cells were washed twice with PBS and lysed by adding 200 μL of lysis buffer to each centrifuge tube, followed by incubation at room temperature for 10 min. An appropriate volume of the cell lysate supernatant was transferred to a new 1.5 mL centrifuge tube, heated at 70 °C for 10 min, and centrifugated at 2000 rpm for 5 min at room temperature. The remaining lysates were used for protein quantification using the BCA protein assay reagent (P1511, Applygen Technologies Inc.). For the TAG assay, 10 μL of the cell lysate supernatant, along with glycerin standards at different dilution levels, was mixed with 190 μL of chromogenic liquid (*n* = 3 per sample). Similarly, 30 μL of the cell-free supernatant, along with glycerin standards, was mixed with 170 μL chromogenic liquid (*n* = 3 per sample). Mixtures were incubated at 37 °C for 15 min before measuring the TAG content. The optical density (OD) at 550 nm, which is proportional to the TAG concentration, was measured using a microplate reader (EPOCH, BioTek Instruments, Inc., Winooski, VT, USA). A standard curve was generated based on the OD values of the glycerin standards, and the TAG content for each sample was calculated accordingly. All measurements were performed in triplicate.

### 2.10. Statistical Analysis

Statistical analysis was performed using GraphPad Prism 9.5.0 software. The significance of differences between pairs of groups was analyzed using Student’s *t*-test. Results are presented as mean ± SEM, with *p*-value < 0.05 considered statistically significant.

## 3. Results

### 3.1. Generation and Verification of TARDBP KO Bovine Mammary Epithelial Cells

**Figure 1:**
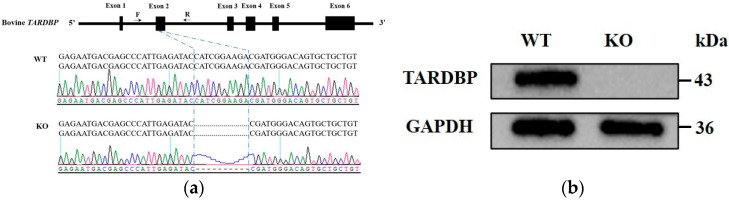
*TARDBP* KO MAC-T cell line was obtained using CRISPR/Cas9 technology. (**a**) Sanger sequencing analyses of the targeted *TARDBP* DNA fragment of both WT and KO cells. The black boxes and the black bold solid lines respectively represent exons and introns of bovine TARDBP gene. The letters F and R represent the forward and reverse primers, respectively. Arrows indicate the direction and approximate location of the primers. Dotted lines represent the position of the missing DNA sequence in the *TARDBP* KO MAC-T cell line compared to that in WT cells. (**b**) Western blot results of Wild-type (WT) and knockout (KO) cells.

To investigate the role of bovine *TARDBP* in milk fat metabolism, a *TARDBP* KO MAC-T cell line was first obtained using CRISPR/Cas9 technology. Sequencing of the genomic region spanning the target site and subsequent sequence alignment analyses revealed a 10 bp deletion (5′-CATCGGAAGA-3′) in exon 2 of the *TARDBP* gene in KO cells, in contrast to WT controls (Figure 1a). This deletion resulted in a frameshift mutation, disrupting the encoding sequence after the 19th amino acid residue and introducing a premature stop codon at the 48th amino acid residue. Western blot analysis further validated the complete absence of *TARDBP* at the protein level in KO cells (Figure 1b). Together, these results confirm the successful establishment of a *TARDBP* KO MAC-T cell model at both the DNA and protein levels.

### 3.2. TARDBP KO Decreases the TAG Content in Both MAC-T Cells and the Supernatant

**Figure 2:**
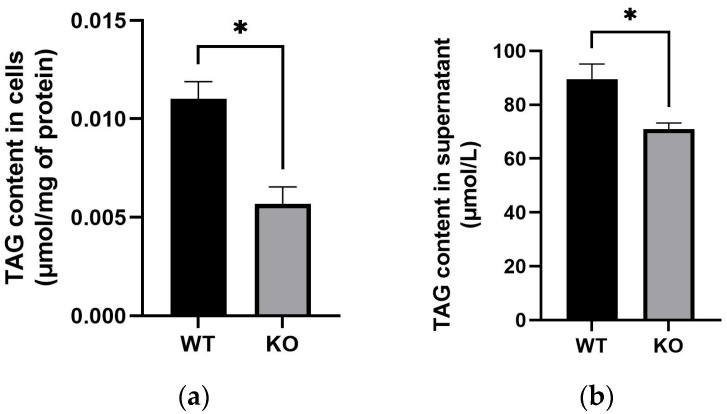
*TARDBP* KO leads to a significant reduction in TAG content in both MAC-T cells and the supernatant. (**a**) Detection of TAG content in WT and KO cells (*n* = 3 per genotype). (**b**) Detection of TAG content in the supernatant from WT and KO cell cultures (*n* = 3 per genotype). * *p*-value < 0.05.

To evaluate the effect of bovine *TARDBP* on milk fat metabolism, TAG content in both MAC-T cells and their culture supernatant was quantified. TAG content was significantly reduced in *TARDBP* KO cells compared to WT controls (Figure 2a; *p*-value < 0.05). Similarly, TAG content in the supernatant of *TARDBP* KO cell cultures was also significantly lower than that in WT cell cultures (Figure 2b; *p*-value < 0.05). These findings demonstrate that *TARDPB* deficiency leads to decreased TAG content both within MAC-T cells and in the extracellular supernatant.

### 3.3. Overview of RNA Sequencing (RNA-Seq) Data

To investigate the impact of *TARDBP* on the transcriptome of MAC-T cells, RNA-seq of both WT and *TARDBP* KO MAC-T cells was carried out. All RNA samples were first assessed for quality, with RNA integrity number (RIN), concentration, and total amount confirming that each sample met the criteria for transcriptome analysis (Table S1). Sequencing quality metrics indicated high data quality, with Q30 scores exceeding 93% for all samples (Table S2) and clean data per sample exceeding 8.16 GB. Mapping statistics showed that more than 91% of clean reads aligned to the bovine reference genome, with over 89% uniquely mapped (Table S3).

**Figure 3:**
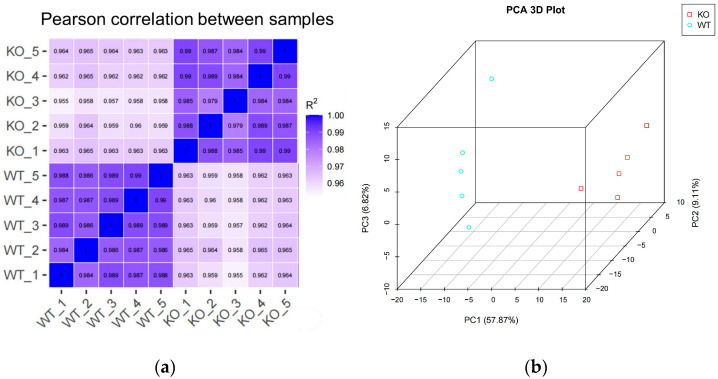
Samples within WT and KO groups show better repeatability and clustering. (**a**) The heat maps for the correlation analysis between samples. R^2^, Pearson correlation coefficient (*n* = 5 per genotype). (**b**) 3D figure for principal component analysis. The horizontal coordinates of the graph are the first principal components, the vertical coordinates are the second principal components, the blue circles represent the WT group and the red squares represent the KO group (*n* = 5 per genotype).

Figure 3a shows that the square of the Pearson correlation coefficient (R^2^) between samples was greater than 0.984 in the WT group and higher than 0.979 in the KO group, indicating strong reproducibility among replicates.

Principal component analysis (PCA), commonly used to assess inter-group variation and intra-group clustering, was carried out based on the FPKM values for each sample. As shown in Figure 3b, the three principal components (PC1, PC2, and PC3) explained 57.87, 9.11, and 6.28% of the total variation, respectively, and effectively distinguished the WT and KO groups. Furthermore, samples within each group clustered closely, demonstrating consistent expression profiles.

### 3.4. Depletion of TARDBP Gene in MAC-T Cells Decreased Expression of CD36, FABP4, DGAT1, PPARG, and PPARGC1A

**Figure 4:**
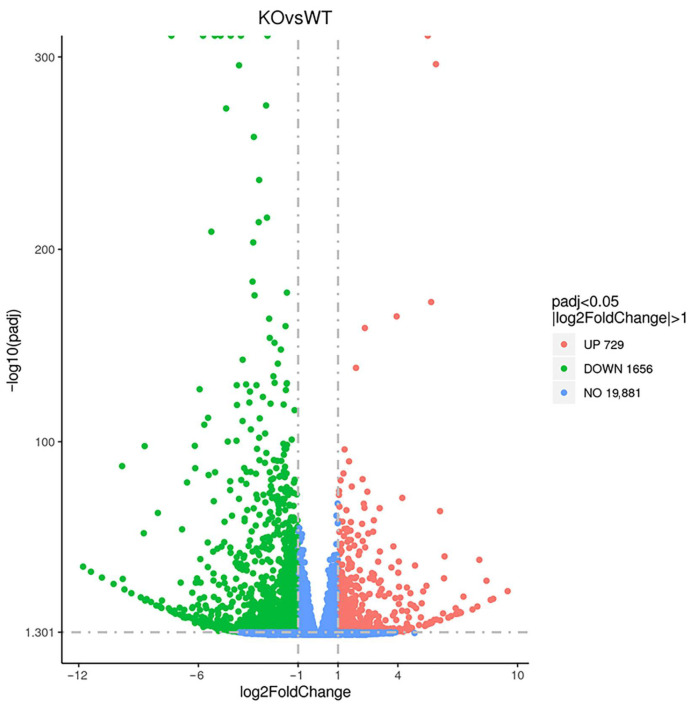
Volcano map for DEGs. The horizontal coordinate is the log2FoldChange value, the vertical coordinate is −log10(padj), and the dashed blue line represents the threshold line of the differential gene screening criteria. The padj represents adjusted *p*-value.

A total of 2385 DEGs were identified between WT and *TARDBP* KO MAC-T cells through transcriptome sequencing. Among these, 729 genes were upregulated, whereas 1656 genes were downregulated in KO cells compared with WT controls (Figure 4). Notably, several key genes involved in milk fat synthesis in mammary epithelial cells—including the fatty acid translocase gene *CD36*, the fatty acid-binding protein gene *FABP4*, the diacylglycerol acyltransferase enzyme gene *DGAT1*, the *PPARG* gene, which encodes the transcription factor that regulates milk lipid synthesis, and its coactivator encoding gene, *PPARGC1A*—were significantly downregulated (Table 2).

### 3.5. Enrichment Analysis of the DEGs

**Figure 5:**
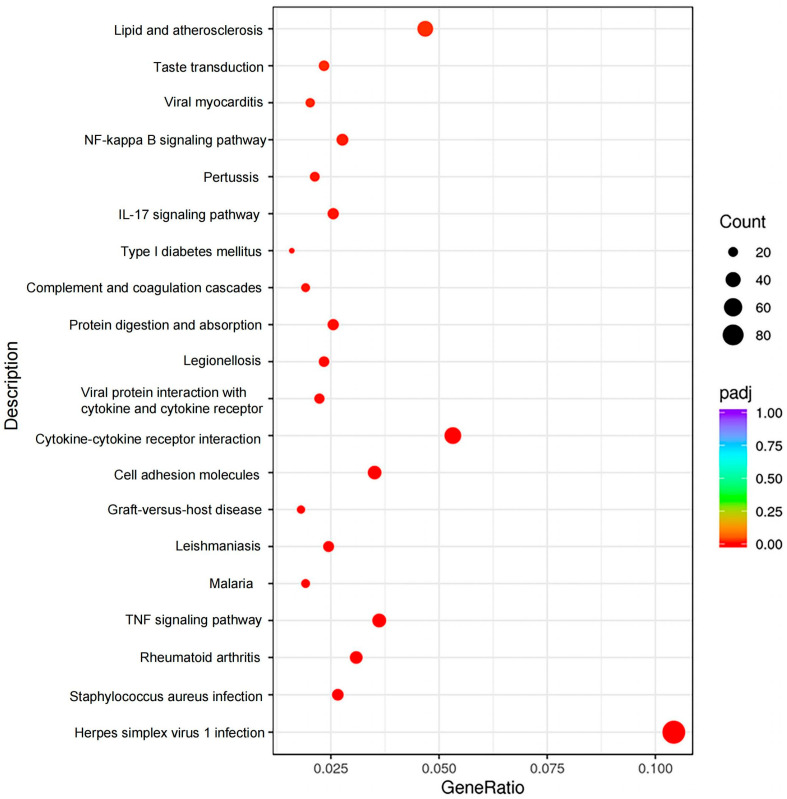
Scatter diagram of KEGG enrichment analysis. The horizontal coordinate is the ratio of the number of differential genes annotated to the KEGG pathway to the total number of differential genes, and the vertical coordinate is the KEGG pathway. Only the top 20 significantly enriched pathways are shown in the diagram. The padj represents adjusted *p*-value.

To further explore the biological functions of the 2385 DEGs, we performed GO and KEGG enrichment analyses. A total of 58 GO terms were significantly enriched (adjusted *p*-value < 0.05), encompassing processes such as immune response, metabolism, development, and others (Table S4). Additionally, KEGG pathway analysis identified 32 significantly enriched pathways (adjusted *p*-value < 0.05), including lipid and atherosclerosis, linoleic acid metabolism, arachidonic acid metabolism, Fc epsilon RI signaling, and others (Figure 5 and Table S5).

### 3.6. The Expression Levels of DEGs Were Verified by qRT-PCR

**Figure 6:**
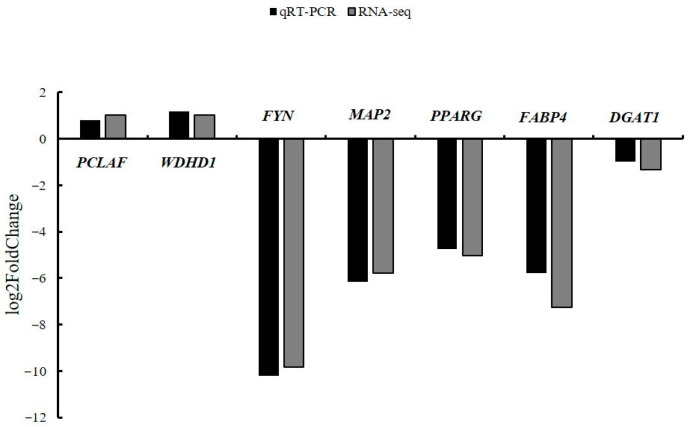
Validation of the DEGs by qRT-PCR. Black: qRT-PCR. Grey: RNA-seq.

Seven DEGs were selected for validation, including four randomly chosen genes and three associated with milk lipid metabolism. Their relative expression levels in WT and *TARDBP* KO cells were quantified by qRT-PCR, and the resulting data were expressed as log2FoldChange. As shown in Figure 6, the qRT-PCR results were consistent with the RNA-seq data, confirming the reliability of the transcriptome analysis.

### 3.7. TARDBP Binding Motif Is Found in DNA Sequences of Both PPARG and PPARGC1A

**Figure 7:**
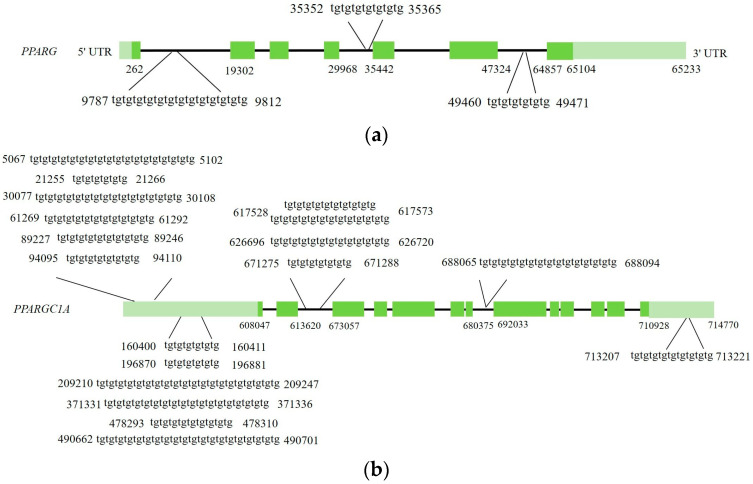
TARDBP binding motif analysis. (**a**) TG-rich sequences in bovine *PPARG* gene; (**b**) TG-rich sequences in bovine *PPARGC1A* gene. Deep green boxes represent coding region. Light green boxes represent non-coding region. Black horizontal lines represent introns.

Sequences of genes showing significantly decreased expression related to milk lipid metabolism—including bovine *PPARG* (NC_037349.1), *PPARGC1A* (NC_037333.1), *FABP4* (NC_037341.1), *DGAT1* (NC_037341.1), and *CD36* (NC_037331.1)—were downloaded from GenBank. As reported in mice, *BTN1A1* and *XDH* contain TARDBP binding motifs. Therefore, the bovine *BTN1A1* (NC_037350.1) and *XDH* (NC_037338.1) sequences were also downloaded at the same time. The presence of TARDBP binding motifs was then analyzed in these genes. As shown in Figure 7, the bovine *PPARG* gene contains TG-enriched sequences within its introns, whereas *PPARGC1A* gene contains numerous TG-rich sequences in its 5′ UTR, 3′ UTR, and introns, suggesting that TARDBP may regulate the mRNA processing or stability of these two genes. However, no TG-rich sequences were identified in the bovine *FABP4*, *DGAT1*, *CD36*, *BTN1A1*, and *XDH* genes.

## 4. Discussion

During lactation of cows, TAG is synthesized in MECs, packaged into lipid droplets, and then secreted into milk. This process of milk fat metabolism encompasses multiple steps, including de novo synthesis of FA, FA uptake, intracellular activation and trafficking, elongation and desaturation, triglyceride synthesis, and lipid secretion [6,7]. Despite extensive research, the gene regulatory networks governing these complex processes remain incompletely elucidated. By binding to UG-rich RNA or TG-rich DNA sequences, TARDBP participates in various molecular processes such as RNA stability, processing, transport, and translation, and DNA transcription, thereby influencing multiple biological functions [14,15,16,17,18,19]. Evidence from studies in mice suggests that *TARDBP* plays a role in fat metabolism [22,23,24]. However, its role in bovine milk fat metabolism has not yet been investigated, and no bovine *TARDBP* KO cell models have been reported to date.

The bovine mammary epithelial cell line MAC-T is capable of synthesizing and secreting milk fat and expresses key genes involved in milk fat metabolism, including *PPARG*, *DGAT1*, *SREBF* and others [37]. In this study, we first generated *TARDBP* KO MAC-T cells using the CRISPR/cas9 technique, providing a valuable model for investigating the role of bovine *TARDBP* in mammary epithelial cells. Using this model, we found that *TARDBP* deletion led to a significant reduction in TAG content in both MAC-T cells and the culture supernatant (*p* < 0.05), which is inconsistent with findings in mice. In mice, TARDBP post-transcriptionally regulates the mRNA stability of *BTN1A1* and *XDH* by binding to UG-rich sequences in their 3′UTR, which are essential for milk lipid secretion of MECs. Consequently, *TARDBP* KO mice exhibit increased intracellular TAG accumulation but reduced milk TAG levels, due to impaired lipid secretion resulting from decreased stability of *BTN1A1* and *XDH* mRNA [24].

To explore the mechanism by which *TARDBP* influences TAG content in MAC-T cells, we conducted transcriptome sequencing of WT and *TARDBP* KO cells, and identified significant downregulation of key genes involved in milk lipid synthesis following *TARDBP* knockout, including *CD36*, *FABP4*, *DGAT1*, *PPARG*, and *PPARGC1A* (adjusted *p*-value < 0.05).

*CD36* encodes a fatty acid translocase that facilitates the uptake of long-chain fatty acids in MECs during lactation [5,38]. Overexpression of *CD36* increases TAG content in bovine MECs, whereas its inhibition significantly reduces both intracellular and extracellular TAG levels [39]. *FABP4* encodes fatty acid-binding protein 4, which mediates intracellular fatty acid transport in MECs [6,7], and has been shown to promote TAG synthesis and lipid droplet accumulation in bovine MECs [12]. *DGAT1* encodes diacylglycerol acyltransferase 1, a key enzyme catalyzing the final step of TAG synthesis [6]. A lysine (K) to alanine (A) substitution in *DGAT1*, caused by an A-A to G-C dinucleotide change in exon 8, has been identified to reduce bovine milk fat content by impairing enzymatic activity critical for TAG synthesis [40,41,42]. *PPARG* encodes the gamma subtype of peroxisome proliferator-activated receptor, a central transcriptional regulator of lipid metabolism in MECs [5,43]; its overexpression significantly enhances lipid accumulation in goat MECs [43]. *PPARGC1A* encodes a transcriptional coactivator that enhances the transcriptional activity of PPARG [44], and its downregulation in bovine MECs decreases intracellular TAG content [45]. Collectively, these genes positively regulate milk fat synthesis. Accordingly, the reduction in TAG content observed in *TARDBP* KO MAC-T cells and their supernatant may result from downregulation of *CD36*, *FABP4*, *DGAT1*, *PPARG,* and *PPARGC1A*. However, these genes were not differentially expressed in RNA-seq data from WT and *TARDBP* KO mouse MECs at lactation onset [21]. Furthermore, milk lipid secretion-related genes *BTN1A1* and *XDH* showed no differential expression between WT and *TARDBP* KO bovine MECs. These findings highlight species-specific differences in the molecular regulation of milk fat metabolism between cattle and mice.

Further analysis of TARDBP binding motifs in the DNA sequences of the aforementioned DEGs related to bovine milk fat metabolism identified TG-rich sequences in the introns of bovine *PPARG* and in the 5′UTR, 3′UTR, and introns of bovine *PPARGC1A*. As anticipated, no TG-rich sequences were detected in bovine *BTN1A1* and *XDH* genes. These findings suggest that TARDBP may regulate RNA processing or stability in bovine cells through binding to UG-rich sequences in *PPARG* and *PPARGC1A* pre-mRNA or mRNA but not in *BTN1A1* or *XDH* mRNA, thereby affecting the mRNA levels of the former two genes. This may be the reason why the TAG content observed from MAC-T cells is inconsistent with that from mouse MECs.

Interestingly, our study did not identify TG-rich sequences in bovine *CD36*, *FABP4*, or *DGAT1*. Nevertheless, these genes are established downstream targets of *PPARG* and/or *PPARGC1A*. Previous studies have demonstrated that *PPARG* positively regulates the expression of *CD36*, *FABP4* and *DGAT1* in buffalo and goat MECs [46,47,48]. Similarly, *PPARGC1A* has been reported to positively regulate *CD36* expression in bovine MECs and *DGAT1* expression in goat MECs [45,49]. These findings suggest that the decreased expression of *CD36*, *FABP4* and *DGAT1* observed in *TARDBP* KO MAC-T cells may be indirectly mediated through the downregulation of *PPARG* and *PPARGC1A*.

Despite providing new insights into the role of *TARDBP* in bovine milk fat metabolism, our study has several limitations. First, the findings were obtained in MAC-T cells, an immortalized bovine mammary epithelial cell line, which may not fully recapitulate the in vivo environment of the bovine mammary gland. Validation in primary bovine mammary epithelial cells and in vivo animal models will be required to confirm the physiological relevance of our results. Second, although we identified TG-rich sequences in PPARG and PPARGC1A and hypothesize that TARDBP may regulate their mRNA stability, direct binding was not experimentally validated in this study. Approaches such as RNA immunoprecipitation (RIP) or crosslinking immunoprecipitation (CLIP) could be used in future studies to confirm TARDBP–RNA interactions. Finally, lipid metabolism is a highly complex process influenced by multiple regulatory pathways, and our study focused on a limited set of genes; thus, additional regulatory targets of TARDBP remain to be elucidated. Addressing these limitations in future research will further clarify the role of TARDBP in bovine milk fat metabolism.

## 5. Conclusions

In summary, we found that *TARDBP* deficiency reduces the milk fat content in bovine mammary epithelial cells and their culture supernatant, as well as the mRNA levels of *CD36*, *FABP4*, *DGAT1*, *PPARG* and *PPARGC1A*. Given the presence of TARDBP binding motifs within the genomic sequences of *PPARG* and *PPARGC1A*, we propose that bovine TARDBP may regulate the processing or stability of *PPARG* and *PPARGC1A* mRNA by binding to their UG-rich sequences, thereby modulating the expression of downstream genes, including *CD36*, *FABP4*, and *DGAT1*, and consequently affecting both intracellular and extracellular TAG content. This study provides novel insights into the role of *TARDBP* in bovine milk fat metabolism and lays a foundation for further investigations into the molecular regulatory mechanisms and genetic improvement of milk fat traits. Nonetheless, further studies are required to elucidate the precise molecular mechanisms by which bovine *TARDBP* affects TAG content in MAC-T cells.

## Figures and Tables

**Table 1:** Sequence information of primers for qRT-PCR.

Primer Name	Primer Sequence (5′–3′)	Product Length/bp
*FABP4*	F: TGAGATTTCCTTCAAATTGGG	101
	R: CTTGTACCAGAGCACCTTCATC	
*DGAT1*	F: GCAACGCACGGTTATTTCT	122
	R: CACAATGACCAGGCACAGAG	
*PPARG*	F: GAGATCACAGAGTACGCCAAG	216
	R: GGGCTCCATAAAGTCACCAA	
*MAP2*	F: AGTAGTCACGGCGGAAGC	145
	R: TTCTGAGGCTGGTGATGG	
*FYN*	F: GGACGGAAGATGACCTGA	156
	R: CTTCTGCCTGGATGGAGT	
*WDHD1*	F: TGGCATCCTACTTGTGGTCA	123
	R: CTTTTCTACTCTGCTAGACACCTTA	
*PCLAF*	F: AGCTACAGAAAAGTGGTGGCT	136
	R: GGCGCACACAAACTGGATTC	
*β-actin*	F: CATCGGCAATGAGCGGTTCC	147
	R: ACCGTGTTGGCGTAGAGGTC	

**Table 2:** The downregulated genes involved in milk lipid metabolism.

Gene ID	Gene Name	Log2FoldChange	Adjusted *p*-Value
281052	*CD36*	−2.1097	4.13 × 10^−5^
281759	*FABP4*	−7.2700	1.81 × 10^−12^
282609	*DGAT1*	−1.3322	6.43 × 10^−33^
281993	*PPARG*	−5.0435	1.18 × 10^−13^
338446	*PPARGC1A*	−2.0187	1.24 × 10^−2^

## Data Availability

The raw data supporting the conclusions of this article will be made available by the authors upon request.

## Supplementary Materials

The following supporting information can be downloaded at: https://www.mdpi.com/article/10.3390/ani15172607/s1, Table S1: Summary of data quality of RNA sequencing for each sample; Table S2: Summary of clean reads mapped to the *Bos taurus* reference genome; Table S3: GO terms with an adjusted *p*-value < 0.05; Table S4: KEGG pathways with an adjusted *p*-value < 0.05; Table S5: KEGG pathways with an adjusted *p*-value 0.05; Figure S1: Sanger sequencing chromatograms for assessing the editing activity of pGK1.1-sgRNA in the bovine *TARDBP* gene; Figure S2: Complete Western blot figures.

## Abbreviations

The following abbreviations are used in this manuscript:

MEC	Mammary Epithelial Cell
TAG	Triacylglyceral
KO	Knockout
WT	Wild-type
UTR	Untranslated Region
RNA-seq	RNA Sequencing
PCA	Principal Component Analysis
PC	Principal Component
DEGs	Differentially Expressed Genes
GO	Gene Ontology
KEGG	Kyoto Encyclopedia of Genes and Genomes
qRT-PCR	Quantitative Real-Time PCR
FA	Fatty acid
RIP	RNA immunoprecipitation
CLIP	Crosslinking immunoprecipitation
