# Supplementary material for: CRISPR/Cas9-Mediated TARDBP Knockout Reduces Triacylglycerol Content and Key Milk Fat Metabolism Gene Expression in MAC-T Cells
Source: Animals (Basel). 2025 Sep 5;15(17):2607. doi: 10.3390/ani15172607 (PMC12427303; doi:10.3390/ani15172607)
Supplement: Supplementary file 1 [file animals-15-02607-s001.zip › animals-3823678-supplementary.pdf]

Supplementary Tables

Table S1. RNA quality control detection results for each sample.

sample	Concentration (ng/μL)	Volum (μL)	Total Amount (μg)	RNA integrity number (RIN)
WT-1	310.00	35.00	10.850	5.70
WT-2	373.00	35.00	13.055	7.70
WT-3	273.00	35.00	9.555	6.10
WT-4	310.00	35.00	10.850	6.40
WT-5	364.00	35.00	12.740	6.10
KO-1	417.00	35.00	14.595	8.50
KO-2	379.00	35.00	13.265	8.70
KO-3	312.00	35.00	10.920	7.80
KO-4	389.00	35.00	13.615	8.50
KO-5	408.00	35.00	14.280	7.80

Table S2. Summary of data quality of RNA sequencing for each sample.

sample	raw reads	raw bases	clean reads	clean bases	Q20	Q30	GC_content
WT-1	54406408	8.16G	50297378	7.54G	97.62	93.47	52.56
WT-2	63250768	9.49G	58618620	8.79G	97.87	94	50.52
WT-3	56081238	8.41G	52115882	7.82G	97.66	93.55	51.66
WT-4	56398804	8.46G	52797548	7.92G	97.76	93.76	50.76
WT-5	63530868	9.53G	58970566	8.85G	97.71	93.65	51.22
KO-1	58485380	8.77G	54731150	8.21G	97.61	93.39	50.62
KO-2	59331884	8.9G	54871256	8.23G	97.77	93.76	50.28
KO-3	62718624	9.41G	58779938	8.82G	97.87	94.01	50.31
KO-4	61479146	9.22G	57582852	8.64G	97.92	94.16	50.58
KO-5	65345534	9.8G	61116086	9.17G	98.03	94.46	51.28

Table S3. Summary of clean reads mapped to the *Bos taurus* reference genome.

sample	total_reads	total_map	unique_map	multi_map
WT-1	50297378	47841493(95.12%)	46660352(92.77%)	1181141(2.35%)
WT-2	58618620	53794930(91.77%)	52494994(89.55%)	1299936(2.22%)
WT-3	52115882	48297042(92.67%)	47082951(90.34%)	1214091(2.33%)
WT-4	52797548	49042789(92.89%)	47821341(90.57%)	1221448(2.31%)
WT-5	58970566	54889071(93.08%)	53466714(90.67%)	1422357(2.41%)
KO-1	54731150	50964334(93.12%)	49804387(91.0%)	1159947(2.12%)
KO-2	54871256	51101356(93.13%)	49968264(91.06%)	1133092(2.07%)
KO-3	58779938	54696231(93.05%)	53373935(90.8%)	1322296(2.25%)
KO-4	57582852	53851476(93.52%)	52595016(91.34%)	1256460(2.18%)
KO-5	61116086	57174635(93.55%)	55816750(91.33%)	1357885(2.22%)

Table S4. GO terms with an adjusted *p*-value < 0.05.

Category	GOID	Description	Adjusted *P*-value
BP	GO:0006955	immune response	0.000512
BP	GO:0045766	positive regulation of angiogenesis	0.002326
BP	GO:0003008	system process	0.002326
BP	GO:0048871	multicellular organismal homeostasis	0.005242
BP	GO:0051239	regulation of multicellular organismal process	0.005242
BP	GO:1904018	positive regulation of vasculature development	0.005253
BP	GO:0006952	defense response	0.005253
BP	GO:0042445	hormone metabolic process	0.005437
BP	GO:0009605	response to external stimulus	0.005621
BP	GO:0051240	positive regulation of multicellular organismal process	0.009519
BP	GO:0001816	cytokine production	0.011457
BP	GO:0034097	response to cytokine	0.014181
BP	GO:0010959	regulation of metal ion transport	0.016721
BP	GO:0002376	immune system process	0.02298
BP	GO:0009653	anatomical structure morphogenesis	0.02298
BP	GO:0030104	water homeostasis	0.02298
BP	GO:0007600	sensory perception	0.024098
BP	GO:0010033	response to organic substance	0.025507
BP	GO:0050793	regulation of developmental process	0.026609
BP	GO:0051049	regulation of transport	0.026609
BP	GO:0001817	regulation of cytokine production	0.026609
BP	GO:0071345	cellular response to cytokine stimulus	0.028939
BP	GO:2000026	regulation of multicellular organismal development	0.032354
BP	GO:0010562	positive regulation of phosphorus metabolic process	0.032354
BP	GO:0045937	positive regulation of phosphate metabolic process	0.032354
BP	GO:0002252	immune effector process	0.032354
BP	GO:0006954	inflammatory response	0.032354
BP	GO:0010817	regulation of hormone levels	0.032354
BP	GO:0048878	chemical homeostasis	0.032354
BP	GO:0032879	regulation of localization	0.032354
BP	GO:0048584	positive regulation of response to stimulus	0.032354
BP	GO:1902533	positive regulation of intracellular signal transduction	0.034749
BP	GO:0035725	sodium ion transmembrane transport	0.037345
BP	GO:0051094	positive regulation of developmental process	0.037702
BP	GO:0009617	response to bacterium	0.042052
BP	GO:0051924	regulation of calcium ion transport	0.042052
BP	GO:0070555	response to interleukin-1	0.042052
BP	GO:0072376	protein activation cascade	0.042052
BP	GO:0043410	positive regulation of MAPK cascade	0.042366
BP	GO:0001934	positive regulation of protein phosphorylation	0.043863
BP	GO:0050891	multicellular organismal water homeostasis	0.046328
BP	GO:0050877	nervous system process	0.047767

BP	GO:0061041	regulation of wound healing	0.048469
BP	GO:0051707	response to other organism	0.04934
BP	GO:0043269	regulation of ion transport	0.04981
CC	GO:0005576	extracellular region	3.38E-11
CC	GO:0044421	extracellular region part	0.000271
CC	GO:0045121	membrane raft	0.000365
CC	GO:0098857	membrane microdomain	0.000365
CC	GO:0098589	membrane region	0.00057
CC	GO:0005615	extracellular space	0.001217
CC	GO:0045177	apical part of cell	0.00882
CC	GO:0016324	apical plasma membrane	0.011761
CC	GO:0005884	actin filament	0.031011
MF	GO:0005102	signaling receptor binding	0.00161
MF	GO:0048018	receptor ligand activity	0.016677
MF	GO:0030545	receptor regulator activity	0.016677
MF	GO:0005126	cytokine receptor binding	0.016783

Table S5. KEGG pathways with an adjusted *p*-value < 0.05.

KEGGID	Description	Adjusted *P*-value
bta05168	Herpes simplex virus 1 infection	4.55E-06
bta05150	Staphylococcus aureus infection	4.55E-06
bta05323	Rheumatoid arthritis	0.000193
bta04668	TNF signaling pathway	0.000211
bta05144	Malaria	0.000323
bta05140	Leishmaniasis	0.001436
bta05332	Graft-versus-host disease	0.001568
bta04514	Cell adhesion molecules	0.001568
bta04060	Cytokine-cytokine receptor interaction	0.001568
bta04061	Viral protein interaction with cytokine and cytokine receptor	0.002853
bta05134	Legionellosis	0.003028
bta04974	Protein digestion and absorption	0.003496
bta04610	Complement and coagulation cascades	0.005714
bta04940	Type I diabetes mellitus	0.005714
bta04657	IL-17 signaling pathway	0.005714
bta05133	Pertussis	0.006593
bta04064	NF-kappa B signaling pathway	0.008834
bta05416	Viral myocarditis	0.013667
bta04742	Taste transduction	0.015304
bta05417	Lipid and atherosclerosis	0.017817
bta05330	Allograft rejection	0.017817
bta00591	Linoleic acid metabolism	0.026718
bta04670	Leukocyte transendothelial migration	0.026718
bta05143	African trypanosomiasis	0.033503
bta04913	Ovarian steroidogenesis	0.036481
bta00830	Retinol metabolism	0.037559
bta04621	NOD-like receptor signaling pathway	0.039962
bta04664	Fc epsilon RI signaling pathway	0.0408
bta04750	Inflammatory mediator regulation of TRP channels	0.0408
bta04640	Hematopoietic cell lineage	0.0408

bta05146	Amoebiasis	0.044031
bta05322	Systemic lupus erythematosus	0.045164

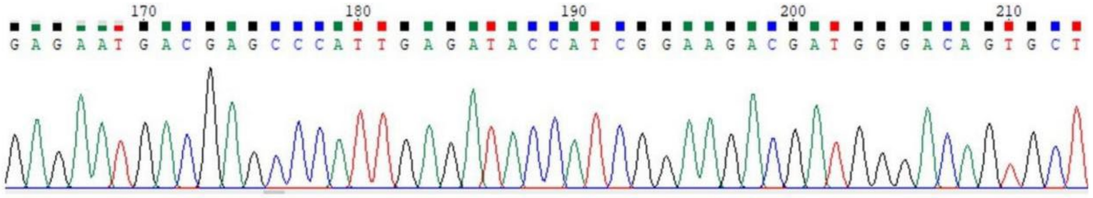

(a)

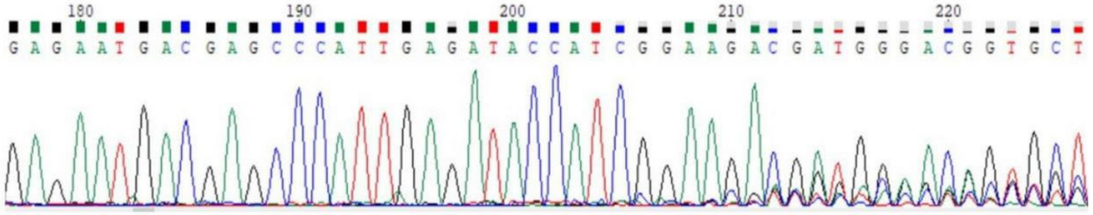

(b)

Figure S1. Sanger sequencing chromatograms for assessing the editing activity of pGK1.1-sgRNA in the bovine *TARDBP* gene. (a) Sequencing chromatogram of the region surrounding the sgRNA-targeted sequence in the bovine *TARDBP* gene from WT MAC-T cells; (b) Sequencing chromatogram of the region surrounding the sgRNA-targeted sequence in the bovine *TARDBP* gene from mixed cells that were transfected with pGK1.1-sgRNA and subjected to drug screening .

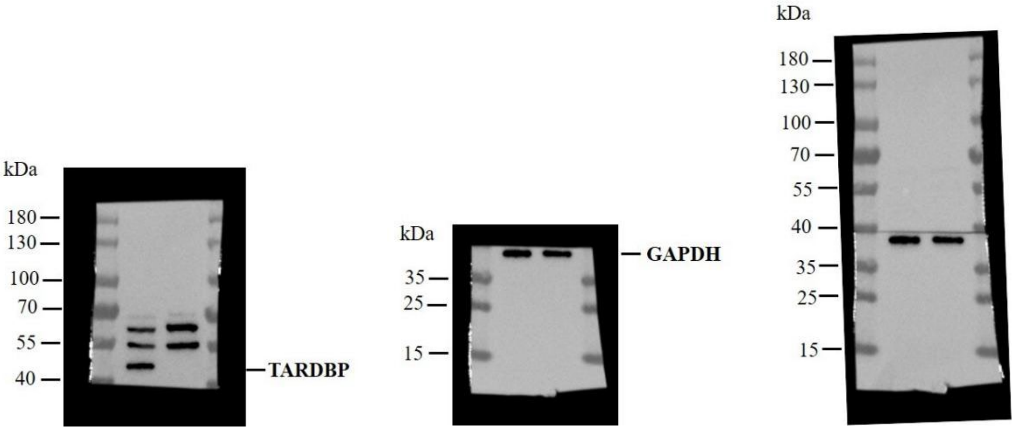

(a) (b) (c)

Figure S2. Complete western blot figures. (a) Single complete western blot figure for TARDBP; (b) Single complete western blot figure for GAPDH; (c) Complete western blot figure for both TARDBP and GAPDH. Note: Because the GAPDH band was brighter than the TARDBP band, and the exposure time was not long enough to reach the required duration for the TARDBP protein, so the TARDBP protein band did not show up clearly in Figure S1c.
